# Silkworm excrement organic fertilizer substitution compound fertilizer improves bamboo shoot yield by altering soil properties and bacterial communities of Moso bamboo *(Phyllostachys edulis)* forests in subtropical China

**DOI:** 10.3389/fpls.2025.1550946

**Published:** 2025-03-17

**Authors:** Zhenya Yang, Jiancheng Zhao, Huijing Ni

**Affiliations:** ^1^ Zhejiang Academy of Forestry, Hangzhou, China; ^2^ Northwest Zhejiang Bamboo Forest Ecosystem Positioning Observation and Research Station, Hangzhou, China

**Keywords:** Moso bamboo, silkworm excrement organic fertilizer, compound fertilizer decrement, bacterial community, soil quality, yield

## Abstract

To achieve high economic benefits, reapplying fertilizers has been a common business measure taken for harvesting Moso bamboo shoots and timber in the past decades in subtropical China. Applying compound and organic fertilizers is an effective measure to enhance soil fertility and promote plant production. To demonstrate how compound fertilizer (CF) decrement and application of silkworm excrement organic fertilizer (SEOF) effect on soil quality, bamboo shoot yield and quality of Moso bamboo plantations, six CF decrement treatments (0 %, 25 %, 50 %, 75 %, and 100 % SEOF substitution, and no fertilization) were examined in our study. Soil nutrients, enzyme activities, bacterial community structures, bamboo shoot yield and quality were determined, and their relationships were analyzed. The results showed that adding SEOF improved soil quality and bamboo shoot yield. Compared with CF, the combined CF-SEOF treatments increased soil pH, soil organic carbon, N and P availability, and the activities of enzymes related to C, N, and P cycling. SEOF substitution significantly changed the soil bacterial community structure and increased the relative abundance of Proteobacteria and Actinobacteria. Higher proportions of organic fertilizer substitution (OF75, OF) enhanced the bamboo shoot yield (by 20.23 % and 16.55 %, respectively) and their total flavonoid and vitamin C content, compared to CF (*p<* 0.05). Moreover, the soil quality index of OF75 and OF50 was significantly higher than that of OF and OF25 in the 0–40 cm soil layer (*p<* 0.05). Pearson’s correlation tests showed that bamboo shoot yield was positively related with soil nutrients (*p<* 0.05). In addition, SEM revealed that fertilization affected soil enzyme activities through soil microorganisms, thereby affecting soil nutrient availability and promoting SQI and bamboo shoot yield. In conclusion, our study revealed that SEOF production is advisable for improving soil quality and bamboo shoot yield, providing evidence that soil nutrients and bacteria contribute to shoot yield and promote the sustainable management of soil and Moso bamboo forests.

## Introduction

Moso bamboo (*Phyllostachys edulis*) is a fast-growing species that is excellent for harvesting bamboo shoots and timber. This forest resource is distributed extensively and covers approximately 73 % of the bamboo acreage in China, significantly influencing the country’s bamboo industry and economic development (Ni and Su, 2024; Song et al., 2011). Moreover, bamboo shoots are widely known for their edible value, great taste, and high nutritional content. It is rich in proteins, vitamins, amino acids, dietary fiber, and flavonoids, making it a staple traditional vegetable in Chinese dishes (Ni et al., 2024a). Intensive management practices, such as cutting and applying large amounts of compound fertilizer (CF) and cutting, have been the main ways to obtain high economic yields of bamboo (Ni et al., 2021). However, the long-term application of chemical fertilizers has resulted in the deterioration of overall soil quality and various problems, such as soil acidification and compaction, increased soil respiration, nutrient depletion, loss of biodiversity, the decline in stand productivity, and ecological environmental damage (Yang et al., 2019; Yao et al., 2022).

In contrast to chemical fertilizers, organic fertilizers are rich in organic matter, nutrients and beneficial microorganisms (Li et al., 2018; Liu et al., 2021; Shao et al., 2024), and they have shown positive effects on soil quality which is typically evaluated using the soil quality index (SQI) (Yang and Zhang, 2023; Ying et al., 2023; Wang et al., 2024). Applying organic fertilizers like manure generally increases the soil organic carbon (SOC) content, nutrient availability and enzymatic activities. It also changes the microbial diversity and composition (Li et al., 2018; Shao et al., 2024). A previous study reported that organic fertilizers can promote nutrient conversion and stimulate plant growth, benefitting crop yields (Oladele et al., 2019). Notably, applying organic fertilizers also affects the nutritional composition of vegetables and fruits (Shankar et al., 2013). A study by Ni et al. (2024b) showed that, compared with CF, humic acid fertilizer increased the SOC and AP content, sucrase (SC) and acid phosphatase (ACP) activities, and bamboo shoot yield in *Phyllostachys violascens* 'Prevernalis' plantations. Applying organic fertilizers is an effective means of decreasing chemical fertilizer input and improving crop yield and soil quality (Ge et al., 2021; He et al., 2022; Ying et al., 2023). Numerous studies have shown that combining CF and organic fertilizers produces the same significant effects. Compared with chemical fertilizers alone (N, P and K), the combined application of inorganic and organic fertilizers can promote the productivity of forestry plants (Liu et al., 2021) and crops like rice, wheat, and corn (Oladele et al., 2019; Shahid et al., 2017; Tang et al., 2018). Combined application can also increase the aboveground biomass of rice (Moe et al., 2019).

Silkworm excrement organic fertilizer (SEOF) is produced by the aerobic and high-temperature composting of silkworm excrement and leftover mulberry leaves, which are fermented by beneficial microorganisms. It is rich in organic matter, N, P, K, and trace elements such as Ca and Mg. It also contains small amounts of alkaloids, various vitamins, and niacin (Chen et al., 2011; Ni et al., 2024c). Its heavy metal and NaCl contents are low, and long-term application cannot easily cause secondary salinization. It has a pH of 8 to 9 and can slow down the progression of soil acidification (Li et al., 2015). It is easily absorbed and utilized by plants, and it can promote plant absorption of N, P, and K, increase their aboveground biomass, and improve crop quality (Luo et al., 2011; Yang et al., 2022). Currently, SEOFs are widely used in the agricultural production of various crops, such as leafy vegetables, peanuts, and corn, increasing soil fertility and crop yield. Its fertilizer efficiency is greater than that of other organic fertilizers like animal manure (chicken, pig, and sheep). For example, compared with chicken manure, SEOF produces higher mass fractions of soil organic matter, total nitrogen (TN), total phosphorus (TP), AN, and AP (Li et al., 2015). SEOF also enhances soil bacterial diversity, promotes the growth of functional and beneficial bacteria, and alleviates obstacles to ensure the continuous cultivation of chrysanthemum (Peng et al., 2015). Moreover, applying SEOF allows the reuse of agricultural and forestry waste, thus reducing environmental pollution and decreasing plant dependence on chemical fertilizers.

The soil is an active site for material cycling and energy exchange in forest ecosystems, providing water and nutrients for plant growth (Li et al., 2023a). Soil quality directly determines plant productivity. Fertilization can alter plant root exudates, microbial growth and reproduction, and soil nutrient retention and mineralization, thereby affecting plant growth (Li et al., 2023b; Yang et al., 2023; Zhou et al., 2015). Soil physical (e.g., bulk density and porosity), chemical (e.g., pH, nutrient content) and biological (e.g., enzyme activities) properties are important components of soil quality (Ning et al., 2020; Wang et al., 2024). Changes in these properties with different fertilization treatments can reflect the sustainability of the soil resource, affecting the yield and quality of bamboo shoots. Previous studies have reported the effects of chemical fertilizers on soil quality and bamboo shoot yield in Moso bamboo forests. Nevertheless, the effects of CF decrement and SEOF application on soil properties and bamboo shoots remain unclear. Thus, we aimed to (1) explore how different percentages of CF combined with SEOF affect soil physicochemical and biological properties; (2) assess the bamboo shoot yield and quality, soil quality index (SQI) in response to fertilization treatments; (3) elucidate the internal links among soil properties, microbial communities, and bamboo shoot yield and quality. The research aimed to provide a theoretical basis for using SEOF to improve soil quality and bamboo yield in Moso bamboo forest sustainable development.

## Materials and methods

### Field site

The research site is located in Maowu Village, Daixi Town (120° 0' 47.3'' E, 30° 39' 33.7'' N), Huzhou City, Zhejiang Province, China, and has an altitude of 65 m. Its climate is categorized under the northern subtropical monsoon climate zone, with an average annual temperature of 12.2–17.3 °C and an average annual precipitation of 1277.6 mm. The Moso bamboo forests were cultivated to enable experimentation, and NPK CF has been applied at a rate of 1500 kg/(ha y) since 2001 to manage the benefits of bamboo shoot production. The forests had a standing bamboo density of 2325–2625 stems/ha and an average diameter at breast height of 10.22–11.59 cm. The soils in the region are classified as red soil. The soil bulk density and pH were 1.24–1.39 g/cm^3^ and 4.36–4.52, respectively.

### Experimental design

In May 2022, six sites were selected for investigation based on their similarities in altitude, slope, and aspect. Thirty 20 m × 20 m plots were divided into six different fertilization treatments, with five replicate plots for each treatment, separated by an interval of 3 m between each plot. In June 2022, sprinkled CF or SEOF was applied to the soil surface, followed by tillage (to a depth of 20–25 cm), and understory vegetation was removed. In August 2023, we dug a ditch 25 cm deep and 15 cm wide, sprinkled CF or SEOF into the ditch, and covered it with soil. The CF had a total nutrient content of ≥ 45 % (N:P_2_O_5_:K_2_O = 20:4:8). The SEOF had a pH of 8.85 and mass fractions of 40 %, 2 %, 1 %, and 2 % for organic matter, N, P, and K, respectively. Based on the proportion of SEOF in the TN input, the treatments were as follows: (1) CF: 100 % CF; (2) OF25: 25 % SEOF and 75 % CF; (3) OF50: 50 % SEOF and 50 % CF; (4) OF75: 75 % SEOF and 25 % CF; (5) OF: 100 % SEOF; and (6) CK: no fertilization. **Table 1** presents the annual fertilization amounts of the experimental Moso bamboo forests.

**Table 1:** Annual fertilization amount used in the experimental forests.

Fertilizer treatments	Annual fertilization amount
CF (100% CF)	1500 kg/ha CF
OF25 (75% CF+25% SEOF)	1125 kg/ha CF+3750 kg/ha SEOF
OF50 (50% CF+50% SEOF)	750 kg/ha CF+7500 kg/ha SEOF
OF75 (25% CF+75% SEOF)	375 kg/ha CF+11250 kg/ha SEOF
OF (100% SEOF)	15000 kg/ha SEOF
CK	No fertilization

### Soil sampling and analysis

In May 2024, soil samples (0–20 cm and 20–40 cm) were collected using a soil drill to collect three soil cores from each plot. The cores were mixed to form one composite sample per plot for a total of 30 composite samples. The fresh soil samples were passed through a 2 mm sieve. A part of the soil was stored at −80°C to extract and determine microbial DNA, and another was collected in a plastic self-sealing bag and air-dried to measure chemical properties and enzyme activities.

Soil pH was determined from a 1:2.5 (V/V) soil-water extract using an electrode pH meter. SOC was measured using a TOC analyzer (Multi N/C 3100; Analytik, Jena, Germany). TN, TP, AN, and AP content were measured using Kjeldahl nitrogen determination method, concentrated H_2_SO_4_ and HClO_4_ digestion method, alkali hydrolyzed diffusion method, and molybdenum antimony colorimetric method, respectively (Ni et al., 2021). Soil urease (UE), SC, and ACP activities were determined with test kits using the 3,5-dinitrosalicylic acid colorimetric method, phenol sodium hypochlorite colorimetric method, and sodium phenylphosphate colorimetric method, respectively (Ni et al., 2024c).

### DNA extraction and high-throughput sequencing

Soil DNA was extracted from a 0.5 g soil sample using a soil DNA Kit (Magen, Guangzhou, China) according to the manufacturer’s protocols. The V3−V4 region was amplified using the following primers: 5′-CCTACGGGNGGCWGCAG-3′ (338F) and 5′-GACTACHVGGGTATCTAATCC-3′ (806R). The 16S rDNA target region of the ribosomal RNA gene were amplified by PCR (95°C for 5 min, followed by 30 cycles at 95°C for 1 min, 60°C for 1 min, and 72°C for 1 min and a final extension at 72°C for 7 min). 50 µL mixture containing 10 µL of 5 × Q5@ Reaction Buffer, 10 µL of 5 × Q5@ High GC Enhancer, 1.5 µL of 2.5 mM dNTPs, 1.5 µL of each primer (10 µM), 0.2 µL of Q5@ High-Fidelity DNA Polymerase, and 50 ng of template DNA. Related PCR reagents were from New England Biolabs, USA. The PCR-purified products from each locus were mixed and subsequently sequenced using Illumina on a NovaSeq 6000 platform at Gene Denovo, Guangzhou, China.

The Amplicon Sequence Variants (ASVs) feature list was generated using the DADA2 (Divisive Amplicon Denoising Algorithm 2) pipeline (Callahan et al., 2016). Subsequently, taxonomic annotations were assigned to the ASVs using the SILVA taxonomy database (Pruesse et al., 2007). Further analyses were conducted using the ASV abundance table after normalization to account for variations in sequencing depth across samples.

### Bamboo shoot yield determination

From March to May 2024, the fresh weight of the bamboo shoots at each sampling site was recorded daily, from the excavation of bamboo shoots to the end of the shoot period. In April 2024, during the peak period of bamboo shoot growth, approximately 5 cm of intact bamboo shoots were randomly excavated from each plot. The shoots were peeled, and 2 kg of the peeled shoots was placed in an icebox and brought back to the laboratory. The samples were crushed and mixed, then used for the determination of total flavonoids and vitamin C content. Total flavonoids content was measured using spectrophotometry and sulfosalicylic acid iron complex colorimetric method. Vitamin C content was determined using test kits.

### Soil quality evaluation

To evaluate the influence of different fertilization treatments on soil quality, the total dataset method (consisting of 12 soil indicators: pH, SOC, TN, TP, AN, AP, SC, UE, ACP, Shannon, Chao1, and ACE) was employed to establish a soil quality index (SQI) for each treatment. The procedure for the calculation is as follows:

All data were standardized and transformed into dimensionless values between 0 and 1 to achieve dimensional normalization of the indicators using the following equations (He et al., 2022):


$$F{\left(Xi\right)}^{'}=\frac{x_{i}-x_{min}}{x_{max}-x_{min}}$$

$$F\left(Xi\right)''=\frac{x_{max}-x_{i}}{x_{max}-x_{min}}$$

where (Xi) , F(Xi)' , and F(Xi)'' is the score of the $i_{th}$ indicator, and $x_{i}$ , $x_{max}$ , and $x_{min}$ are the measured, maximum, and minimum values of the $i_{th}$ indicator, respectively.

Principal component analysis was performed on standard deviation data using SPSS 20.0 (SPSS Inc., Chicago, IL, USA); the common factor variance of soil quality indicators was calculated, and the proportion of the common factor variance of each indicator to the total common factor variance was used as the weight of each indicator.

The SQI was calculated with the equation:


$$\text{SQI}=\sum^{}Wi\times F\left(Xi\right)$$

where Wi the weight of the $i_{th}$ indicator, and F(Xi) the membership value of the $i_{th}$ indicator.

### Statistical analysis

All statistical analyses were performed using SPSS and Excel 2016. One-way analysis of variance followed by Duncan’s multiple range test was used to detect significant differences (*p<* 0.05) between the treatment means. Pearson’s correlation and redundancy analyses were conducted to examine the links between soil chemical properties, bacterial communities, and bamboo shoot yield. Structural equation modeling (SEM) was conducted to examine the relationships among soil properties, enzymatic activities, bacterial diversity, SQI, and bamboo shoot yield. Data from the CF, OF25, OF50, OF75, and OF treatments were used to construct the SEM. All figures were created using Origin 2024 software and R package.

## Results

### Soil chemical properties and enzyme activities

**Table 2** presents the chemical properties of the soil from bamboo forests treated with different fertilizers. The pH, SOC, TN, TP, AN, and AP content from the six treatments showed various trends. Compared with CK, soil pH was significantly higher in OF25, OF50, OF75, and OF by 1.86 %, 3.94 %, 4.87 %, and 4.41 % in the 0–20 cm soil layer, respectively, and by 2.63 %, 9.79 %, 10.26 %, and 14.08 % in the 20–40 cm soil layer, respectively. The SOC contents of OF25, OF50, OF75, and OF were significantly higher than those of CK and CF in the 0–40 cm soil layer. The order of SOC content in the six treatments was OF > OF75 > OF50 > OF25 > CK > CF. The TN, TP, and AN content were significantly higher in OF50, OF75, and OF than in CK and CF. The AP content was significantly higher in OF25, OF50, OF75, and OF than in CF. Compared with CF, OF50, OF75, OF significantly increased the C/N in the 0–40 cm soil layer.

**Table 2:** Soil chemical properties in Moso bamboo forests treated with different fertilizers.

Treatments	pH	SOC	TN	TP	AN	AP	C/N
		g/kg	g/kg	g/kg	mg/kg	mg/kg	
0−20 cm							
CF	4.04 ± 0.03e	10.42 ± 0.30e	1.04 ± 0.02c	0.41 ± 0.02d	104.42 ± 0.60b	76.54 ± 0.10d	10.02 ± 0.21d
OF25	4.30 ± 0.03c	11.73 ± 0.33d	1.06 ± 0.01c	0.66 ± 0.02c	100.15 ± 1.07b	147.32 ± 1.88b	11.07 ± 0.14c
OF50	4.60 ± 0.04b	13.02 ± 0.14c	1.13 ± 0.01b	0.77 ± 0.05a	143.82 ± 0.92a	151.94 ± 3.10b	11.52 ± 0.23c
OF75	4.62 ± 0.04b	20.14 ± 0.30b	1.59 ± 0.00a	0.79 ± 0.03b	148.37 ± 0.88a	173.54 ± 0.35a	12.67 ± 0.15b
OF	4.78 ± 0.02a	21.90 ± 0.59a	1.57 ± 0.03a	0.75 ± 0.04b	122.58 ± 0.54c	129.61 ± 0.29c	13.95 ± 0.26a
CK	4.19 ± 0.03d	10.79 ± 0.53e	0.90 ± 0.04d	0.40 ± 0.04d	85.94 ± 1.62c	73.46 ± 1.02d	11.99 ± 0.32c
20−40 cm							
CF	4.32 ± 0.03d	9.03 ± 0.31e	0.73 ± 0.01c	0.27 ± 0.02c	82.10 ± 0.93b	10.16 ± 0.29c	12.37 ± 0.18c
OF25	4.39 ± 0.02c	10.62 ± 0.18d	0.87 ± 0.01b	0.46 ± 0.03a	95.93 ± 1.95a	57.86 ± 2.21a	12.21 ± 0.27c
OF50	4.48 ± 0.02b	13.79 ± 0.33c	1.01 ± 0.01a	0.43 ± 0.04a	92.59 ± 0.71a	54.32 ± 1.66a	13.65 ± 0.25b
OF75	4.52 ± 0.02a	15.02 ± 0.48b	0.98 ± 0.01a	0.44 ± 0.03a	93.34 ± 0.62a	55.71 ± 1.11a	15.33 ± 0.34a
OF	4.50 ± 0.04a	15.27 ± 0.43a	1.02 ± 0.04a	0.38 ± 0.01b	81.09 ± 0.46b	43.04 ± 1.75b	14.97 ± 0.28a
CK	4.31 ± 0.01d	9.08 ± 0.09e	0.72 ± 0.01c	0.27 ± 0.01c	72.61 ± 1.57c	17.11 ± 1.45c	12.61 ± 0.15c

Different lowercase letters indicate significant differences (*p* < 0.05) between treatments.

**Figure 1:**
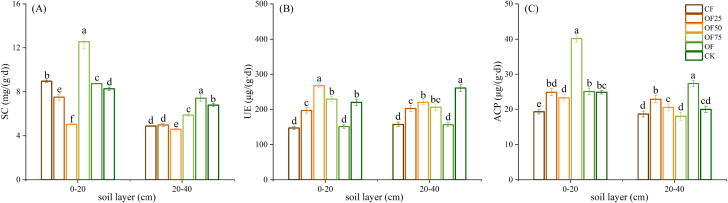
Impact of different fertilization treatments on soil **(A)** sucrase (SC), **(B)** urease (UE), and **(C)** acid phosphatase activities (ACP) in 0−20 cm and 20−40 cm soil layers. Different lowercase letters indicate significant differences (*p*< 0.05) between fertilizer treatments.

Different fertilizer treatments had significant effects (*p<* 0.05) on the soil enzyme activity in each soil layer (**Figure 1**). Compared with CF, the following were observed: OF25, OF75, and OF had increased SC activity in the 0–40 cm soil layer; OF25, OF50, and OF75 had increased UE activity in the 0–20 cm soil layer; and OF25, OF50, and OF had increased ACP activity in the 0–40 cm soil layer. The SC and ACP activities of OF75 were highest in the 0–20 cm soil layer, and those of OF were highest in the 20–40 cm soil layer.

### Soil bacterial community structure

Statistical analysis of high-throughput sequencing results revealed a total of 81 930 ASVs (ranging from 5795−9091 per sample) assigned with a similarity level of ≥ 97 % found across the 60 soil samples (**Table 3**). The alpha diversity indices for community richness (ACE and Chao1) and community diversity (Shannon) in the soil varied among treatments. In the 0–20 cm soil layer, the Shannon index of CF was significantly higher than that of CK but did not differ significantly from those of the other four fertilizer treatments (OF25, OF50, OF75, and OF). In the 20–40 cm soil layer, the Shannon index of OF50 was significantly higher than those of the other treatments. The ACE and Chao1 indices of OF50 were significantly higher than those OF and CK in the 0–20 cm soil layer and significantly higher than those of all other treatments in the 20–40 cm soil layer.

**Table 3:** Sequence statistics and alpha diversity indices of soil bacteria in Moso bamboo forests treated with different fertilizers.

Treatments	ASVs	Shannon	Chao1	ACE	Good Coverage
0−20 cm					
CF	7447 ± 685ab	11.84 ± 0.12a	7447 ± 685ab	7447 ± 685ab	1.00 ± 0.00
OF25	7085 ± 1226ab	11.49 ± 0.20ab	7085 ± 1226ab	7085 ± 1226ab	1.00 ± 0.00
OF50	8112 ± 1187a	11.77 ± 0.30ab	8112 ± 1187a	8112 ± 1187a	1.00 ± 0.00
OF75	7242 ± 780ab	11.54 ± 0.24ab	7242 ± 780ab	7242 ± 780ab	1.00 ± 0.00
OF	6436 ± 296b	11.47 ± 0.07ab	6436 ± 296b	6436 ± 296b	1.00 ± 0.00
CK	6241 ± 963b	11.41 ± 0.25b	6241 ± 963b	6241 ± 963b	1.00 ± 0.00
20−40 cm					
CF	6532 ± 1404b	11.44 ± 0.33b	6532 ± 1404b	6532 ± 1404b	1.00 ± 0.00
OF25	5851 ± 684b	11.17 ± 0.12b	5851 ± 684b	5851 ± 684b	1.00 ± 0.00
OF50	9091 ± 723a	11.96 ± 0.08a	9091 ± 723a	8091 ± 723a	1.00 ± 0.00
OF75	6377 ± 1713b	11.37 ± 0.39b	6377 ± 1713b	6377 ± 1713b	1.00 ± 0.00
OF	6721 ± 1316b	11.55 ± 0.30b	6721 ± 1316b	6721 ± 1316b	1.00 ± 0.00
CK	5795 ± 1056b	11.13 ± 0.29b	5795 ± 1056b	5795 ± 1056b	1.00 ± 0.00

**Figure 2:**
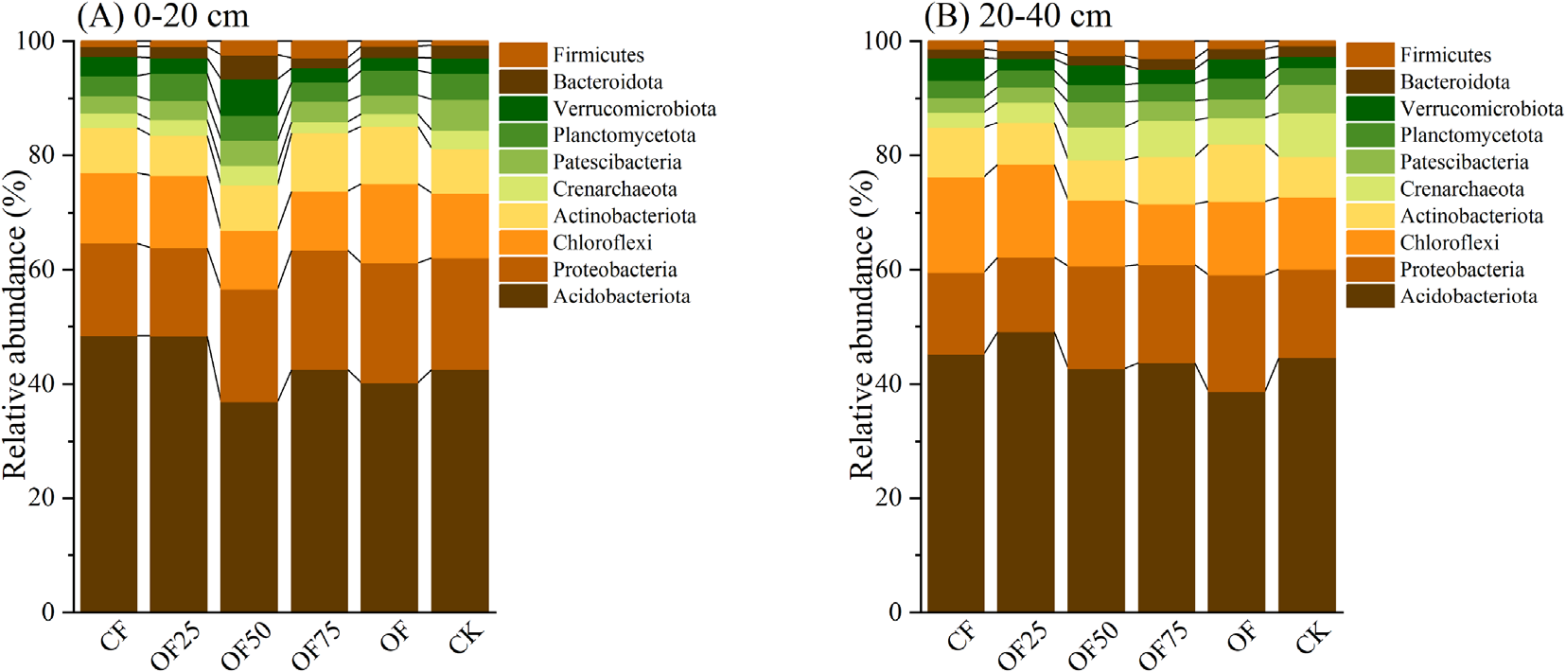
Species composition of soil bacteria at the phylum level in **(A)** 0-20 cm and **(B)** 20-40 cm soil layers in Moso bamboo forests treated with different fertilizers.

**Figure 3:**
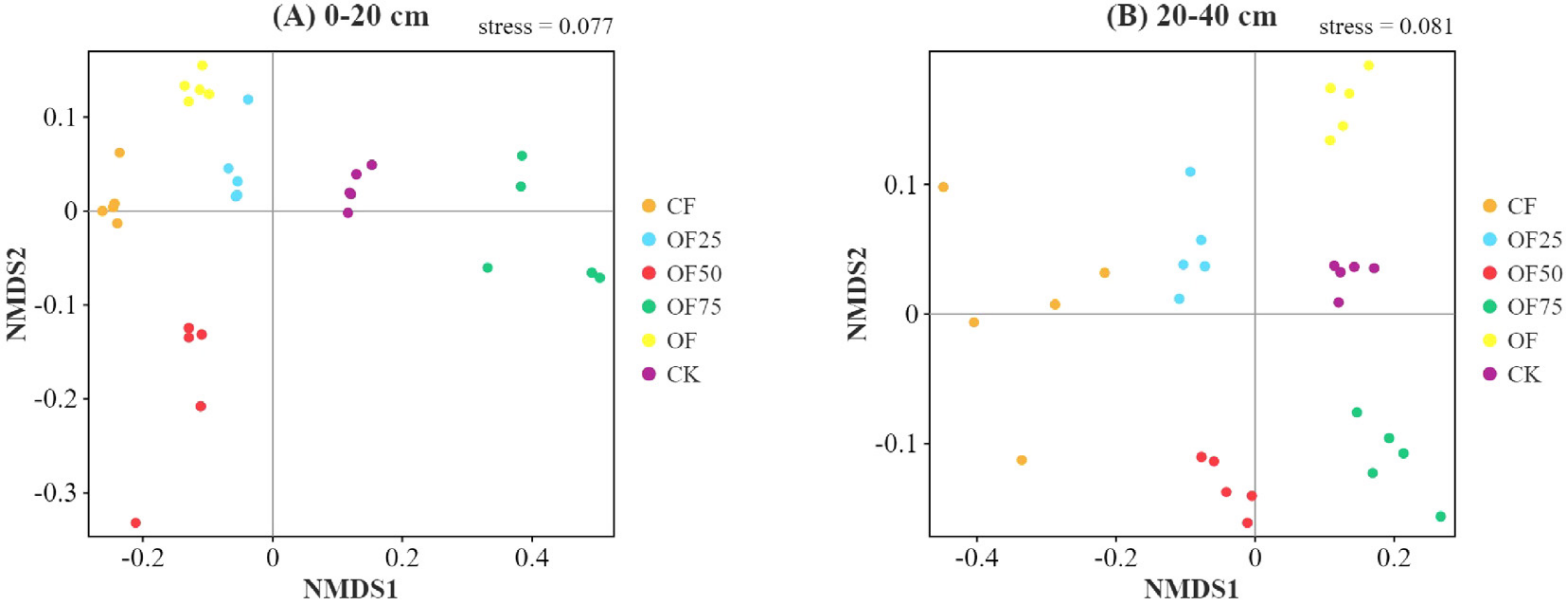
Changes in soil bacterial beta diversity in **(A)** 0-20 cm and **(B)** 20-40 cm soil layers in based on the Bray-Curtis distance Moso bamboo forests treated with different fertilizers at the ASV level.

**Figure 2** shows the most abundant bacterial phylum in all samples. During the entire incubation period, Acidobacteria, Proteobacteria, Chloroflexi, and Actinobacteria were the most dominant phyla across all treatments, accounting for 28.74 %, 10.43 %, 8.02 %, and 5.36 % of the population in the 0–20 cm soil layer, respectively, and 39.04 %, 16.45 %, 12.86 %, and 8.01 % in the 20–40 cm soil layer, respectively. The relative abundance of Proteobacteria in the 0–40 cm soil layer was significantly higher in OF50, OF75, and OF than in CF. The relative abundance of Acidobacteria was lower in OF50, OF75, and OF than in CF and OF25. Based on the Bray–Curtis distance, non-metric multidimensional scaling analysis revealed significant differences in the bacterial community composition at the ASV level (**Figure 3**).

### Bamboo shoot yield and quality

**Figure 4:**
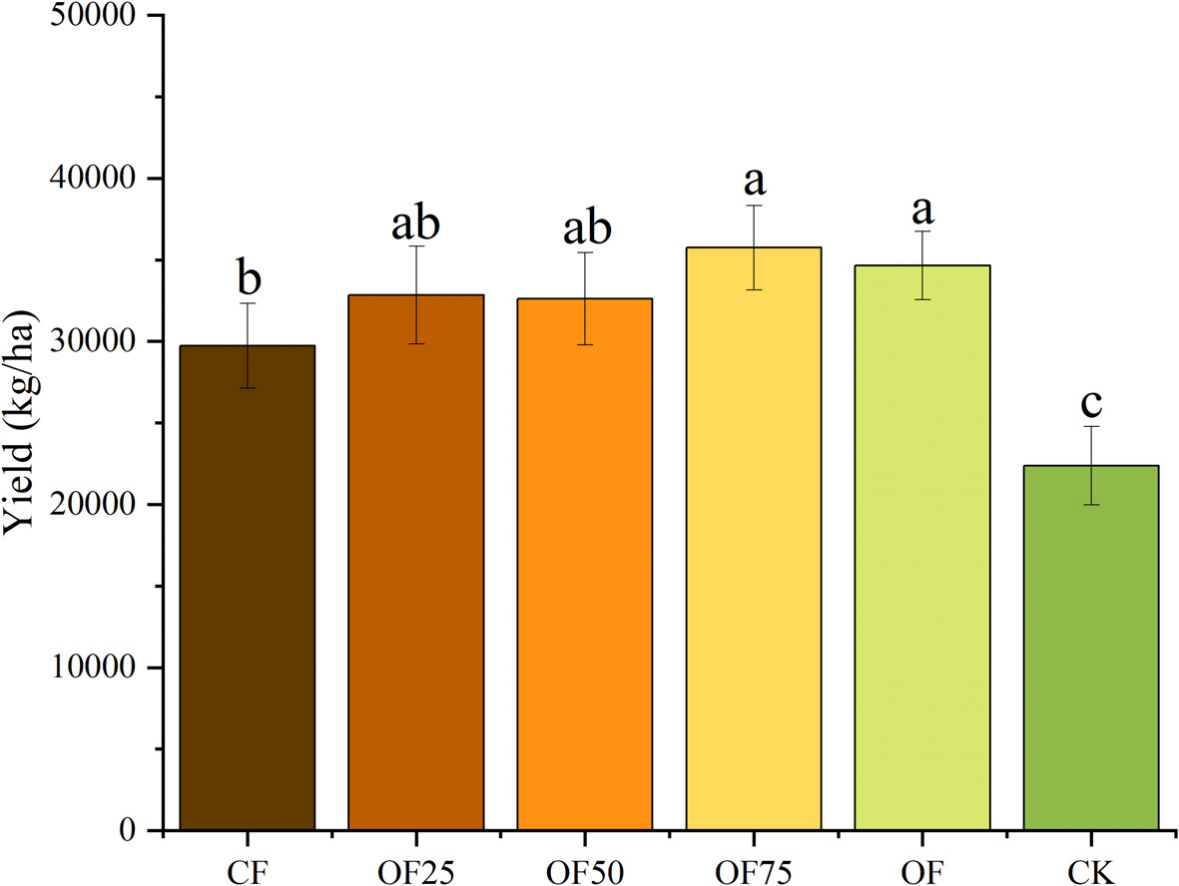
Changes in bamboo shoot yield under different fertilization treatments. Different lowercase letters indicate significant differences (p < 0.05) between treatments.

**Figure 5:**
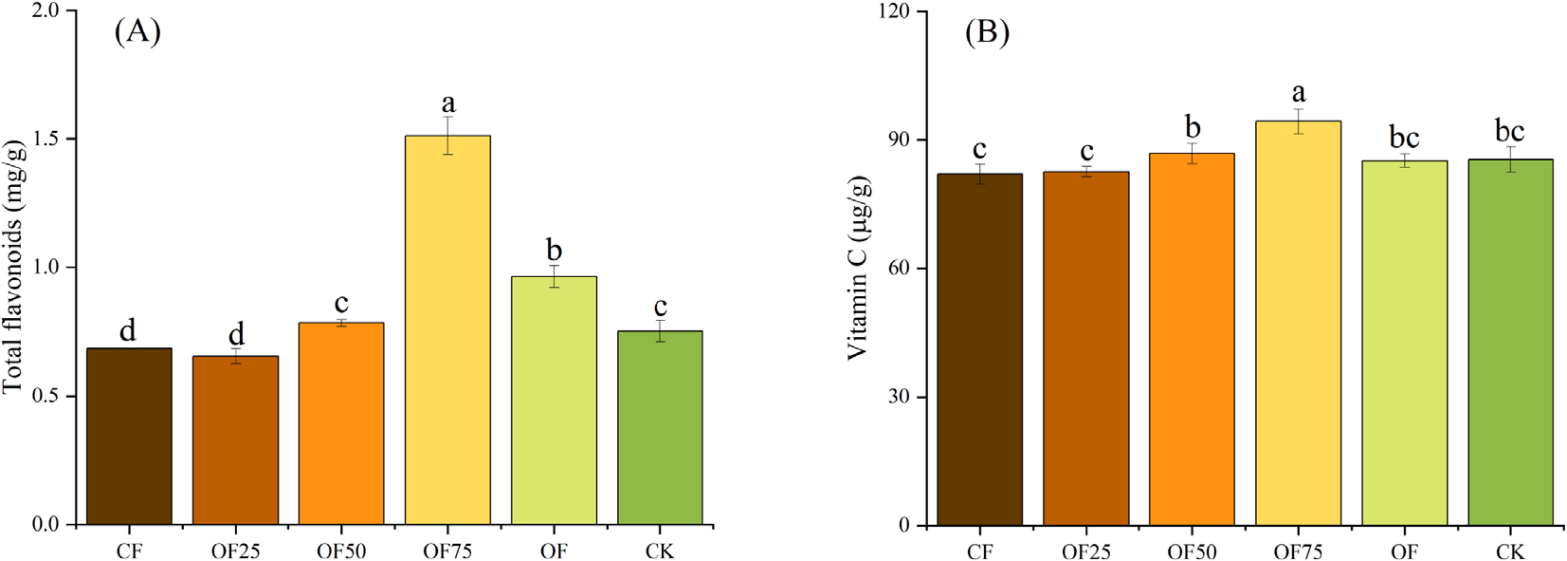
The effects of different fertilization treatments on the nutritional qualities [**(A)** Total flavonoids, **(B)** Vitamin C] of bamboo shoots. Different lowercase letters indicate significant differences (p < 0.05) between treatments.

After two years of fertilization, the bamboo yields of OF75 and OF were 35 745 and 34 650 kg/ha, respectively (**Figure 4**). Compared with CF and CK, OF75 increased the yield by 20.23 % and 59.72 %, respectively, and OF increased the yield by 16.55 % and 54.83 %, respectively (*p<* 0.05). Compared with CF and CK, OF75 significantly increased the total flavonoid content by 122.1 % and 101.3 %, respectively, whereas OF increased it by 41.2 % and 28.0 %, respectively (**Figure 5**). OF50 and OF75 significantly increased the vitamin C content by 5.8 % and 15.0 %, respectively, compared with CF.

### Relationships among soil properties, enzyme activities, bacterial community structure, and bamboo shoot yield and quality

**Figure 6:**
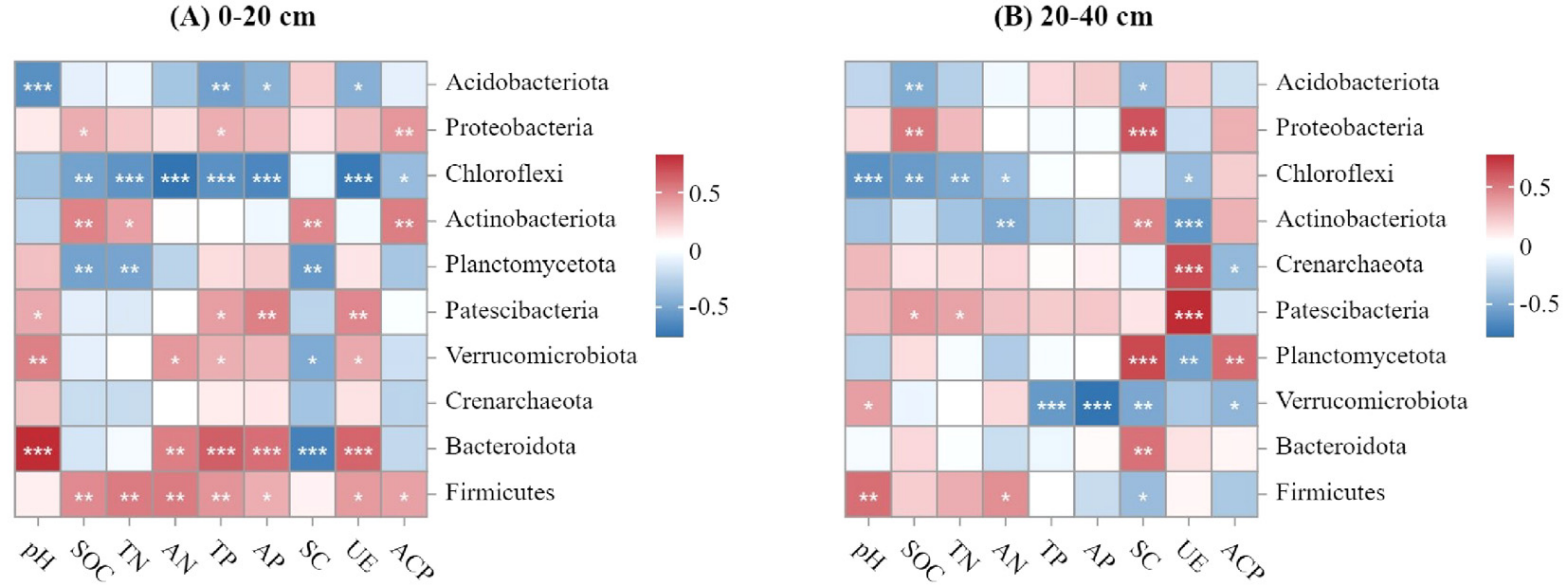
Correlation heatmap of soil bacterial community compositions (phylum level) and soil properties in **(A)** 0-20 cm and **(B)** 20-40 cm soil layers in Moso bamboo forests. (*p<0.05, **p<0.01, ***p<0.001).

**Figure 7:**
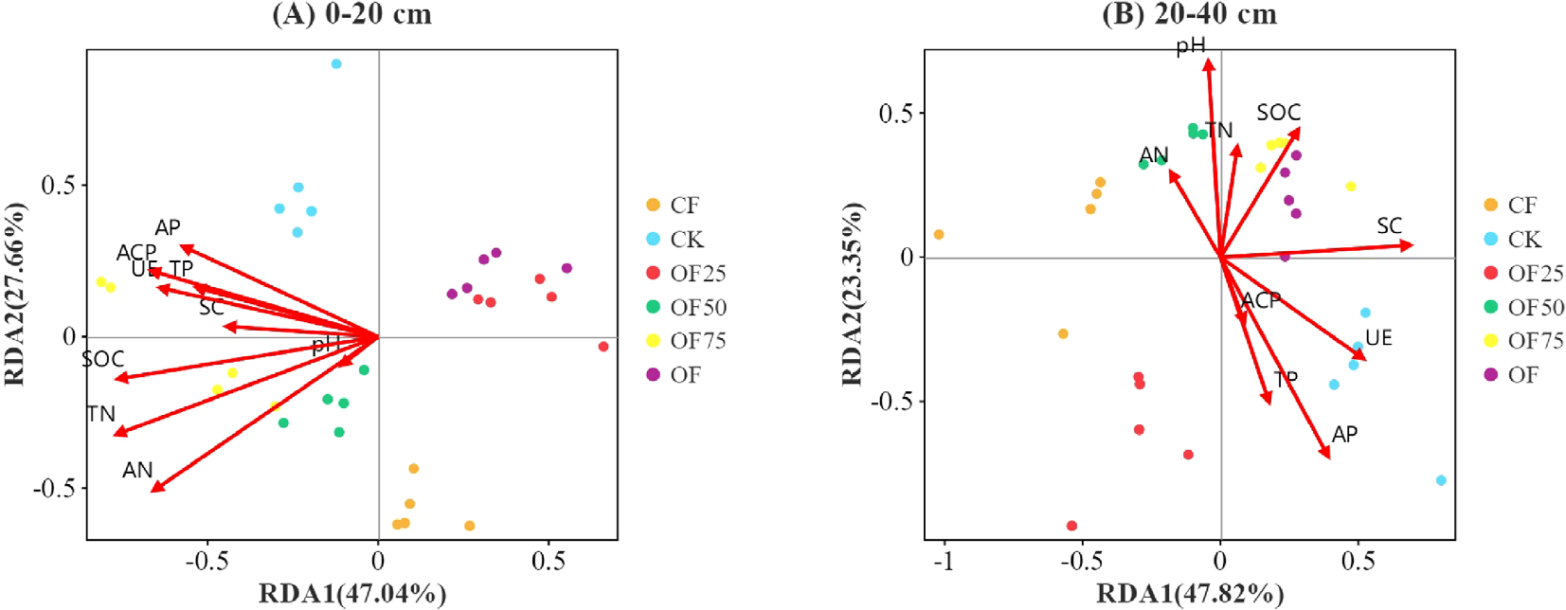
Redundancy analysis (RDA) of soil bacterial community (ASV level) and soil properties for individual samples in **(A)** 0-20 cm and **(B)** 20-40 cm soil layers.

The correlation heatmap (**Figure 6**) shows the relationships between soil bacterial community composition (phylum level) and the main soil characteristics. SOC, TN, and ACP were positively correlated with Firmicutes and Actinobacteria. In the 0–20 cm soil layer, AN, TP, AP, and UE were positively correlated with Bacteroidetes, whereas SOC, TN, AN, TP, AP, UE, and ACP were negatively correlated with Chloroflexi. In the redundancy analysis, the first two axes explained 47.04 % and 27.66 %, 47.82 % and 23.35 % of the variation observed in the microbial communities in the 0–20 cm and 20–40 cm soil layer, respectively (**Figure 7**). In the 0–40 cm soil layer, SOC, TP, AP, and UE had significant effects on the soil bacterial communities (**Table 4**).

**Table 4:** Environmental factors affecting soil bacterial communities in Moso bamboo forests.

Envfit	R^2^	*p−*value	R^2^	*p−*value
	0−20 cm		20−40 cm	
pH	0.0168	0.8	0.4041	0.003
SOC	0.5302	0.001	0.2409	0.018
TN	0.622	0.001	0.1287	0.165
AN	0.6293	0.001	0.1009	0.248
TP	0.2612	0.015	0.2428	0.029
AP	0.348	0.005	0.5475	0.002
SC	0.1682	0.074	0.4188	0.002
UE	0.3692	0.005	0.3401	0.003
ACP	0.4137	0.001	0.0474	0.506

**Figure 8:**
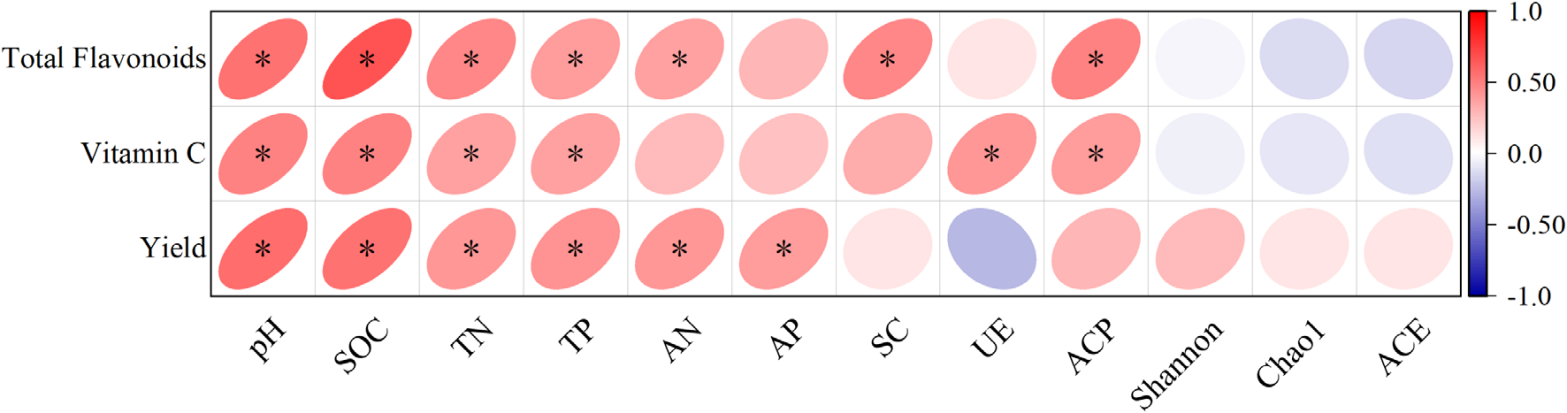
Pearson’s correlation for testing the association among bamboo shoot qualities, yield and soil properties in Moso bamboo forests treated with different fertilizers. (*p<0.05).

Pearson’s correlation tests were performed to examine whether variations in the bamboo shoots were influenced by soil properties (**Figure 8**). Total flavonoid and vitamin C content were positively correlated with pH, SOC, TN and TP. Shoot yield was positively correlated with pH, SOC, TN, TP, AN and AP.

### Soil quality evaluation and its correlation with bamboo shoot yield

**Figure 9:**
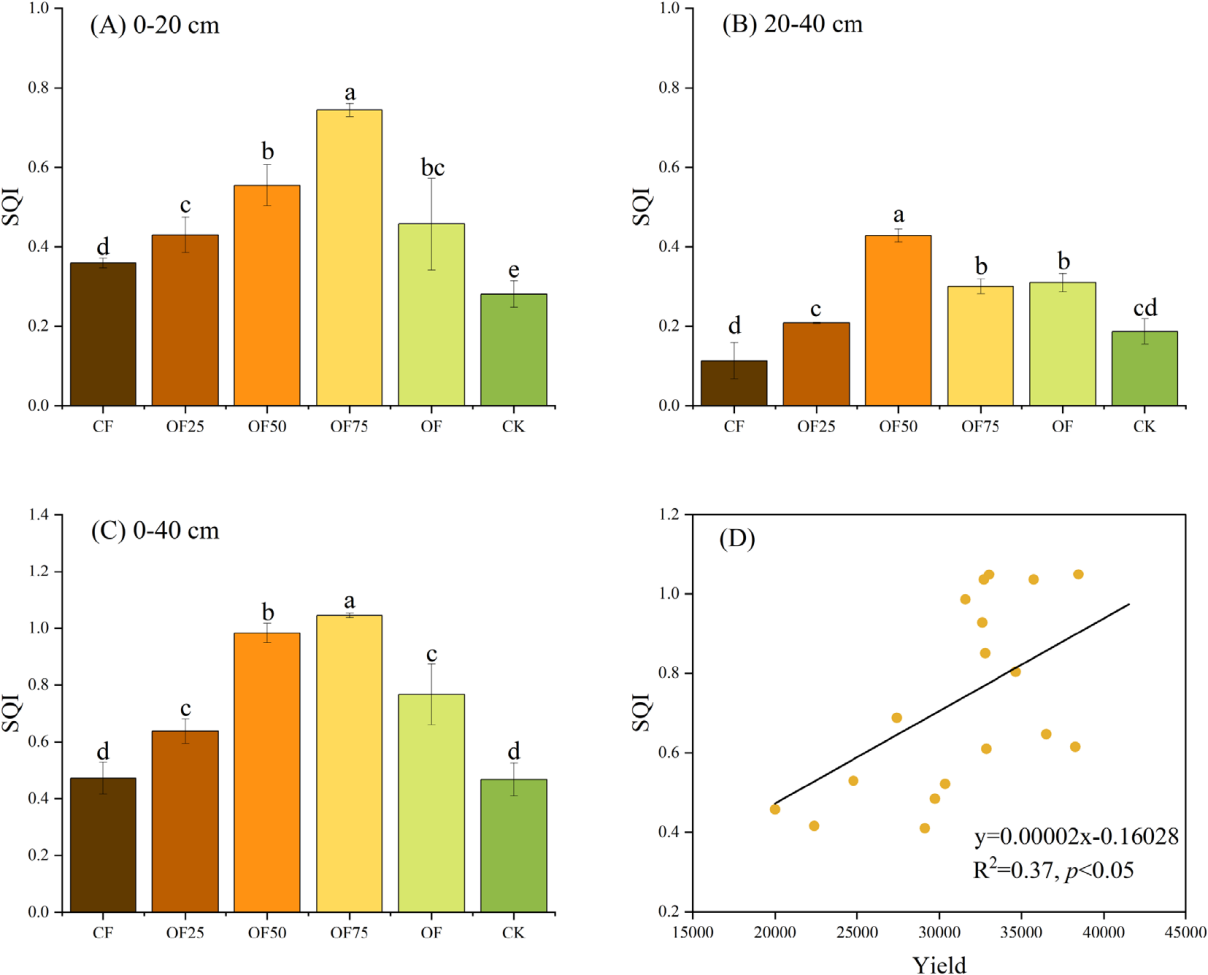
Effects of different fertilizer treatments on soil quality index (SQI, **(A)** 0–20 cm, **(B)** 20–40 cm, **(C)** 0–40 cm) and its correlation with bamboo shoot yield **(D)**. Different lowercase letters indicate significant differences (p < 0.05) between treatments.

The SQI of the six treatments followed the order OF75 > OF50 > OF > OF25 > CF > CK in the 0–20 cm soil layer and OF50 > OF > OF75 > OF25 > CK > CF in the 20–40 cm soil layer (**Figures 9A, B**). Moreover, in the 0–40 cm soil layer, SQI was significantly higher in OF50 and OF75 than that in CF and CK (**Figure 9C**). Correlation analysis showed a significant positive correlation between SQI and bamboo yield (*p*< 0.05, **Figure 9D**).

**Figure 10:**
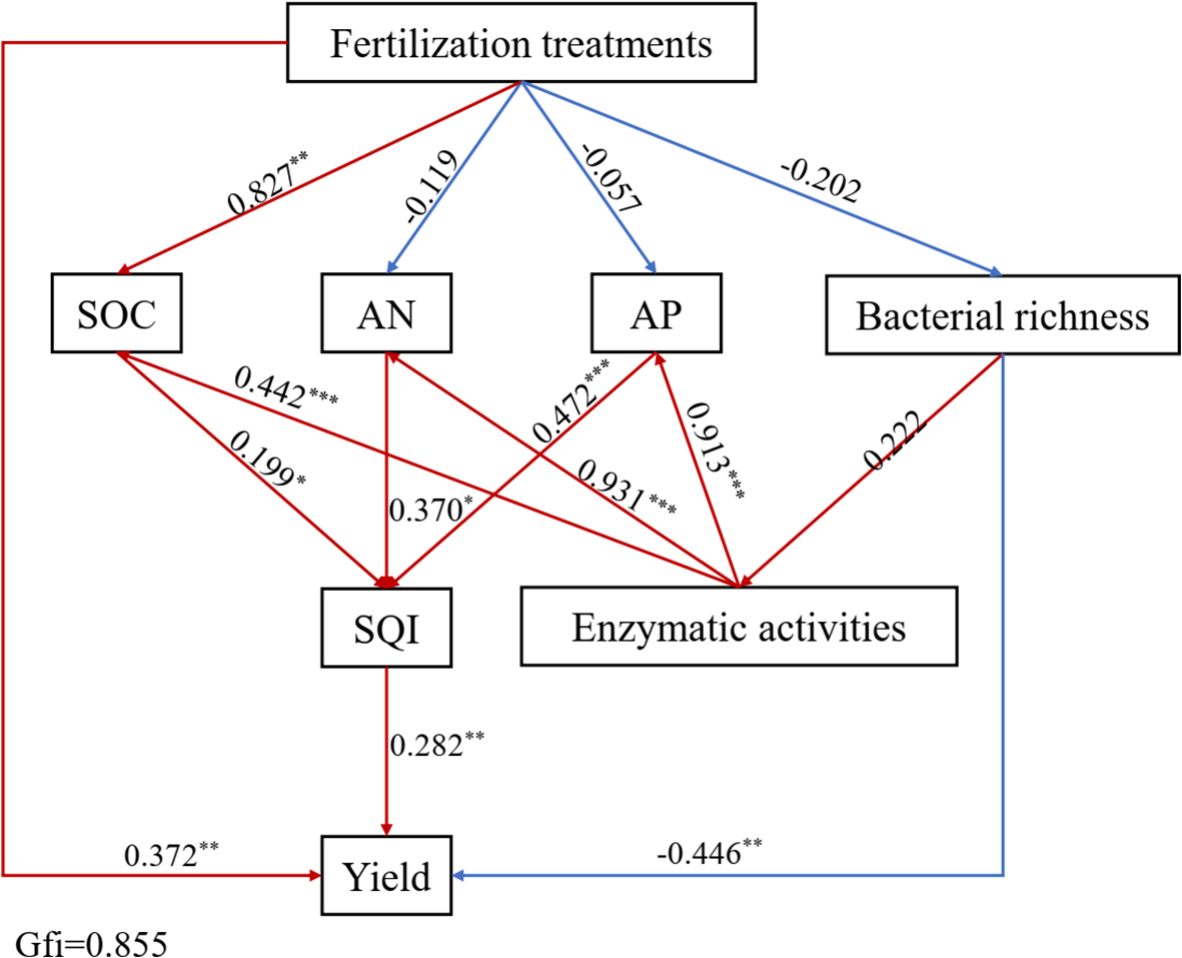
Structural equation modeling (SEM) analysis performed to evaluate pathways of fertilizer treatments influence on the bamboo yield, soil properties, enzymatic activities, bacterial diversity and SQI in the 0–40 cm soil layer. Red and blue arrows indicate significantly (*p*<0.05) positive and negative relationships, respectively. Numbers on arrows represent standardized path coefficients. (*p<0.05, **p<0.01, ***p<0.001).

SEM analysis showed that the fertilization treatments had direct and indirect effects on soil properties, SQI, and bamboo shoot yield (GFI = 0.855, df =28, **Figure 10**). The fertilization treatments had positive and indirect effects on SQI by positively affecting SOC and negatively affecting AN and AP. Additionally, the fertilization treatments had negative effects on AN, AP, and bacterial diversity. Bacterial diversity had a positive effect on enzymatic activities (SC, UE, and ACP), which had direct significant positive effects on SOC (*p*< 0.001), AN (*p*< 0.001), and AP (*p*< 0.001). Finally, SQI had a significant positive effect on bamboo shoot yield (*p*< 0.01).

## Discussion

### Impacts of different fertilization treatments on soil chemical properties

Our results indicated that after adding different SEOF treatments (OF25, OF50, OF75, and OF) to the soil for two years, the soil pH increased compared with that of CK (**Table 2**) because SEOF is alkaline and can reduce soil acidification. Zhang et al. (2019) reported that soil acidification is more effectively alleviated by applying high proportions of organic fertilizer than by applying lower proportions. The SOC content of OF50, OF75, and OF were significantly higher than those of CK and CF in the 0–20 cm and 20–40 cm soil layers, respectively. Possible reasons for the increase in soil C stocks are as follows: first, the C content of the SEOF used in this experiment was 40 %; and second, the input of organic carbon and organic matter from the SEOF increased microbial activity and metabolites which is beneficial for the conversion of exogenous carbon into SOC, further promoting the accumulation of SOC (Wang et al., 2015). The combination of chemical and organic fertilizers can increase crop biomass and the input of fresh organic matter into the soil and crop root exudates, further improving the accumulation and fixation of SOC (Yu et al., 2012).

Decreasing the application of CF and applying organic fertilizer as an alternative increased the soil nutrient availability, which is consistent with the results of previous studies (Chen et al., 2020; He et al., 2022; Luo et al., 2018; Ying et al., 2023; Lu et al., 2024). In this study, SEOF effectively improved soil nutrient availability, as shown by the increased levels of soil nutrients (e.g., TN, TP, AN, and AP) compared with CK and CF (**Table 2**). Similarly, previous studies have reported that a combination of chemical and organic fertilizers improves soil fertility (Li et al., 2023b; Liu et al., 2021). The increased availability of N and P in the soil is partly due to the slow release of nutrients from organic fertilizers. Organic acids generated during organic material decomposition can promote nutrient release, thereby improving the effectiveness of soil nutrients (Cheng et al., 2024). In addition, the large amount of organic matter input from organic fertilizers increases the availability of substrates for microorganisms, which enhances microbial activity, accelerates the decomposition of animal and plant residues, converts soil organic N into inorganic N, and releases effective nutrients for plant utilization (Ning et al., 2017). Applying organic fertilizers also promotes the activation of inorganic P in the soil and increases the AP content (Ying et al., 2023).

### Impacts of different fertilization treatments on soil enzyme activities

Soil enzymes are indicators of soil quality and are mainly derived from soil microorganisms, animal and plant residues, and organic fertilizers (Ni et al., 2021). Soil enzyme activities are closely related to SOC decomposition, nutrient mineralization, and plant nutrient absorption and utilization, and they are highly sensitive to external environmental changes (Ni and Su, 2024). Applying SEOF increased soil SC activity, which catalyzes the hydrolysis of sucrose into fructose and glucose for plant absorption and utilization. SC also participates in the mineralization of organic carbon, and its activity is closely related to SOC content (Ni et al., 2024b). The SC and ACP activities of OF75 were highest in the 0–20 cm soil layer, which indicates that applying a combined fertilizer with a high proportion of organic fertilizer improved soil enzyme activities. Phosphatases are important, because they provide P for plant uptake by releasing phosphate ion from immobile organic P (Liu et al., 2010). Urease catalyzes the hydrolysis of urea to CO_2_ and NH_3_. UE activity was significantly higher in OF25, OF50, and OF75 than in CF in the 0−40 cm soil layer (**Figure 1**), indicating that applying a certain amount of SEOF promoted the hydrolysis of urea, which in turn improves soil N availability and N supply capacity. Similar results have also been reported by other researches, who found organic fertilizer had strongly effect on the urease activity (Saha et al., 2008; Liu et al., 2010).

The increase in soil enzyme activity after organic fertilizer treatment may be because of the increased concentrations of organic matter. In addition, the increase in nutrient availability can also stimulate microbial growth (Zhang et al., 2019). On the other hand, the decrease in soil enzyme activity after CF treatment may be because of the changes in the microbial community structure caused by the acidification induced by chemical fertilizers (Zhang et al., 2019). Another study also reported that adding chemical fertilizer negatively influenced both acid and alkaline phosphatase activities (Saha et al., 2008), which is consistent with our findings.

### Impacts of different fertilization treatments on soil bacterial community structures

Microbial community structures respond to different fertilization treatments. Our study showed that adding fertilizer significantly changed the α−diversity of the soil bacterial community based on the community indices. In the 0–40 cm soil layer, the ACE and Chao1 indices of OF50 were significantly higher than those OF and CK, indicating that apply 50% organic substitution improved the bacterial community richness. The enhanced richness of bacterial under organic and compound fertilization was consistent with other papers (Hartmann et al., 2015), and confirmed that variation of community mainly attributed to quality of the organic fertilizers. These results are similar to the studies, which showed that fertilization can directly enrich specific microbial communities or indirectly affect bacterial composition by altering soil characteristics (Guo et al., 2018).

The main bacterial phyla in all treatments were Acidobacteria, Proteobacteria, Chloroflexi, and Actinobacteria (**Figure 2**), which is similar to the results of a previous study (Ni et al., 2024c). This is mainly because a large amount of nutrient input can stimulate the growth of microorganisms in the soil (Ying et al., 2023). However, in our study, the relative abundance of Acidobacteria decreased, and that of Proteobacteria increased after treatment with a higher proportion of organic fertilizer. Some studies have demonstrated that adding organic fertilizer primarily decreases oligotrophic taxa, such as Chloroflexi and Acidobacteria, and increases copiotrophic taxa, such as Bacteroidetes and Proteobacteria (Li et al., 2021a; Zeng et al., 2016). These changes are consistent with the copiotrophic hypothesis, which states that under nutrient-rich conditions, faster-growing copiotrophic taxa increase whereas slower-growing oligotrophic taxa decline (Fierer et al., 2012; Ying et al., 2023). In addition, Actinobacteria enriched the soil after organic fertilizer treatment because they rapidly decompose organic matter and convert it into available nutrients (N, P, and K) that plants can use, thereby providing nutrients for plant growth (Ni et al., 2024c; Yang et al., 2023). Therefore, we speculate that applying organic fertilizers may affect the bacterial community structure through various soil factors, such as soil pH, enzyme activity, and nutrient bioavailability.

### Bamboo shoot yield and quality and their relationship with soil quality

In our study, OF75 and OF increased the bamboo shoot yield by 20.23 % and 16.55 %, respectively, compared with CF (**Figure 4**). This indicates that adding a certain amount of SEOF increased the yield of bamboo shoots. This is consistent with previous studies, which demonstrated that adding organic fertilizer can increase crop yield (He et al., 2022; Liu et al., 2021; Ni et al., 2024b). Organic fertilizer improved soil structure, capacity to preserve moisture and fertility, and microbial activity, thereby providing better growth conditions for crops (Liu et al., 2010; Liu et al., 2022b). In addition, the process of slow decomposition and nutrient release of organic materials provides more continuous nutrient supply for crop growth (Gai et al., 2018; Li et al., 2021b). Fertilization enhances plant growth and development and improves plant quality by increasing nutrients like vitamin C and total flavonoids (He et al., 2022). In our study, applying organic fertilizer (OF75, OF) significantly increased the total flavonoid and vitamin C content of bamboo shoots and improved their nutritional value compared with applying CF alone.

Several workers had shown that environmental factors significantly impact the abundance, diversity, and function of soil microorganisms (Ge et al., 2021; Yang et al., 2023). In this study, redundancy analysis showed that SOC, TP, AP, and UE were strongly correlated with soil bacterial communities (**Figure 7**, **Table 4**). In the 20–40 cm soil layer, the correlation heatmap showed a significant positive correlation between Acidobacteria, Proteobacteria, and SOC (**Figure 6**). Acidobacteria and Proteobacteria are primarily involved in organic matter decomposition and biological N fixation. Chloroflexi is a bacterial phylum that generates energy through photosynthesis and can break down polysaccharides in soil into organic acids and hydrogen, thereby promoting the degradation of organic matter and cellulose (Ni et al., 2024c). The positive links among SOC, TN, AN, UE, and Chloroflexi demonstrate that Chloroflexi is closely related to C cycling and bamboo forest productivity.

The SQI constructed from multiple soil properties can reflect the effect of fertilization on soil quality (Li et al., 2020; Chen et al., 2021; He et al., 2022). Our study showed that the SQI of OF75 was highest, and followed by OF50 in the 0–40 cm soil layer. All treatments involving the organic fertilizers showed an increased SQI, indicating that any combination of reduced compound fertilizer containing organic fertilizer was superior to a single compound fertilizer. This agrees with the works that in D’Hose et al. (2014). We found that bamboo shoot yield was positively correlated with soil pH, SOC, N, P and SQI (**Figures 9**, **10**), indicating that soil pH and fertility levels affected shoot yield and that yield changes with soil quality. This may be because applying organic fertilizers alleviated soil acidification by providing the nutrients required for bamboo shoot growth and development. Similarly, some studies have reported increased yields of wheat and rice (He et al., 2022; Liu et al., 2022a). Additionally, organic fertilizers have a long-term effect on maintaining soil fertility and the sustainable management of bamboo forests (Li et al., 2023b). Fertilization can be expected to increased plant growth either directly or indirectly via influence the soil nutrients, activities and microbial communities. Our SEM analysis showed that fertilization treatments had a positive and indirect effect on SQI by positively affecting SOC and that SQI had a positive effect on bamboo yield (**Figure 10**). These results demonstrate that decreasing CF and applying organic fertilizer as an alternative increased bamboo shoot yield by improving soil quality. This agrees with previous studies reporting that improving soil quality is beneficial for increasing crop yields (wheat and maize) (Li et al., 2020; Pan et al., 2020). Li et al. (2021b) reported that organic fertilizer addition increased yield and soil quality compared with adding chemical fertilizers alone in a double cropping system in China, which is consistent with our findings. Moreover, fertilization promoted enzyme activity by affecting bacterial diversity, thereby enhancing soil quality and bamboo shoot yield (**Figure 10**). These results indicate that the changes in soil nutrient status and microbial community caused by organic fertilizers are closely related and may work together to improve yield (Liu et al., 2021).

The impact of organic fertilizer application on soil improvement and bamboo shoot yield is a long-term and slow process. This experiment was only conducted for 2 years, and long-term monitoring experiments should be conducted in the future to more comprehensively evaluate the effect of applying organic fertilizer on soil quality, shoot yield, and shoot quality in Moso bamboo forests.

## Conclusions

Our study highlighted that adding organic fertilizers improved the quality of the soil and the yield of the bamboo shoots. Compared with CF addition alone, the combined application of CF and organic fertilizer increased soil pH, SOC, N, and P availability, and the activities of enzymes related to C, N, and P cycling. Organic fertilizer substitution also significantly changed the soil bacterial community structure and increased the relative abundance of beneficial bacteria, such as Proteobacteria and Actinobacteria. Higher proportions of organic fertilizer substitution (OF75, OF) enhanced the bamboo shoot yield (by 20.23 % and 16.55 %, respectively) and their total flavonoid and vitamin C content, compared to CF. In addition, SEM revealed that fertilization affected soil enzyme activities through soil microorganisms, thereby affecting soil nutrient availability and promoting SQI and bamboo shoot yield. Moreover, the SQI of OF75 and OF50 was significantly higher than that of OF and OF25 in the 0–40 cm soil layer. In conclusion, our study revealed that SEOF production is advisable for improving soil quality and bamboo shoot yield.

## Data Availability

The raw data supporting the conclusions of this article will be made available by the authors, without undue reservation.
